# Fumarate hydratase-deficient renal cell carcinoma: a case report and review of the literature

**DOI:** 10.1186/s13256-023-03841-0

**Published:** 2023-04-20

**Authors:** Yanting Lv, Lide Song, Mengjun Hu

**Affiliations:** 1 Department of Pathology, Zhuji People’s Hospital, Shaoxing, Zhejiang People’s Republic of China; 2 Department of Urology, Zhuji People’s Hospital, Shaoxing, Zhejiang People’s Republic of China

**Keywords:** Fumarate hydratase, Renal cell carcinoma

## Abstract

**Background:**

Fumarate hydratase-deficient renal cell carcinoma is a rare pathological subtype that was defined by the World Health Organization (WHO 5th edition) in 2022. At present, only a few hundreds of cases have been reported worldwide, mainly in Europe and the United States. A case of a Chinese patient is reported here, along with a literature review.

**Case report:**

A 60-year-old Asian male who complained of hematuria for 20 days was admitted to the hospital. Contrast enhanced Computer Tomography showed that the volume of the right kidney was increased, with a patchy low-density shadow with infiltrative growth inside that had a significantly lower signal intensity than the renal cortex; thus, the possibility of collecting duct carcinoma or lymphoma, was considered. Enlarged perirenal and retroperitoneal lymph nodes were also seen, along with bilateral renal cysts. Eight years prior, ultrasonography had shown a complex renal cyst in the right kidney, and no treatment was administered at that time. Laparoscopic radical nephrectomy of the right kidney was performed this time, and the postoperative specimens were submitted for pathological examination. Because immunohistochemistry showed the loss of fumarate hydratase protein expression and the possibility of fumarate hydratase-deficient renal cell carcinoma was considered, corresponding molecular pathological tests were performed, and the results showed an FHp.R233H (arginine > histidine) germline mutation (inactivation mutation). The postoperative pathological diagnosis was fumarate hydratase-deficient renal cell carcinoma in the right kidney, T3aN1M0. The patient was treated with sunitinib, and bone and liver metastases developed half a year later. The treatment was then changed to axitinib and toripalimab. At present, the patient is in stable condition, and there has been no progression of the metastases.

**Conclusion:**

Fumarate hydratase-deficient renal cell carcinoma is a very rare renal tumor that is defined on a molecular basis. It is highly malignant and metastasizes early. Therefore, fully understanding the disease, enabling detection and diagnosis and administering treatment are particularly important.

## Background

Fumarate hydratase-deficient renal cell carcinoma is a rare pathological subtype caused by a pathogenic mutation in the fumarate hydratase gene located in 1q42.3-q43 and was defined by the World Health Organization (WHO 5th edition) in 2022 [1]. This type of renal cell carcinoma is highly invasive and can metastasize when the tumor volume is very small [2]. Due to its rarity and the lack of understanding of its clinical manifestations, the exact incidence of the disease is unknown. The literature shows that the disease occurs all over the world, but it is mainly seen in Europe and the United States [3]. The incidence of fumarate hydratase-deficient renal cell carcinoma is rare in Asian populations, especially in Chinese populations. Thus, this report presents a case of a rare form of this malignancy, fumarate hydratase-deficient renal cell carcinoma; because of the rarity of this disease, this article can be a valuable source of information for other medical professionals facing similar conditions. Moreover, this is an intriguing case of a very rare renal tumor that was defined on a molecular basis, and its characteristics will be thoroughly discussed below.

## Case report

**Fig. 1:**
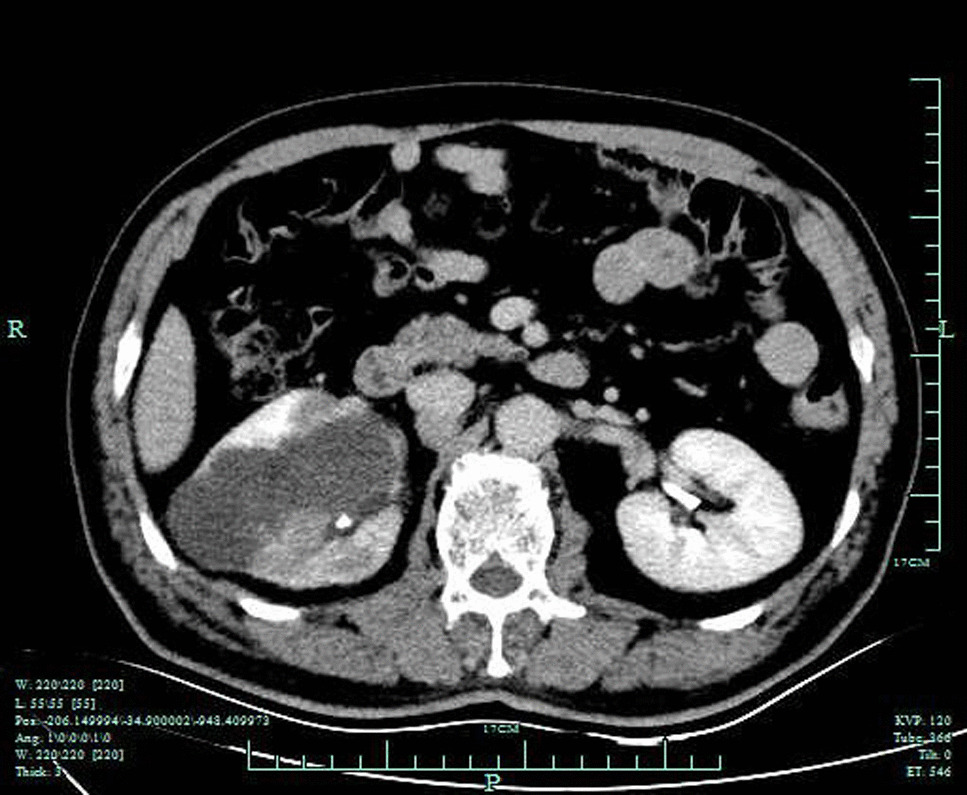
Contrast enhanced Computer Tomography showing that the volume of the right kidney was increased, with a patchy low-density shadow with infiltrative growth inside that had significantly lower signal intensity than that the renal cortex. Enlarged lymph nodes can be seen around the kidney

**Fig. 2:**
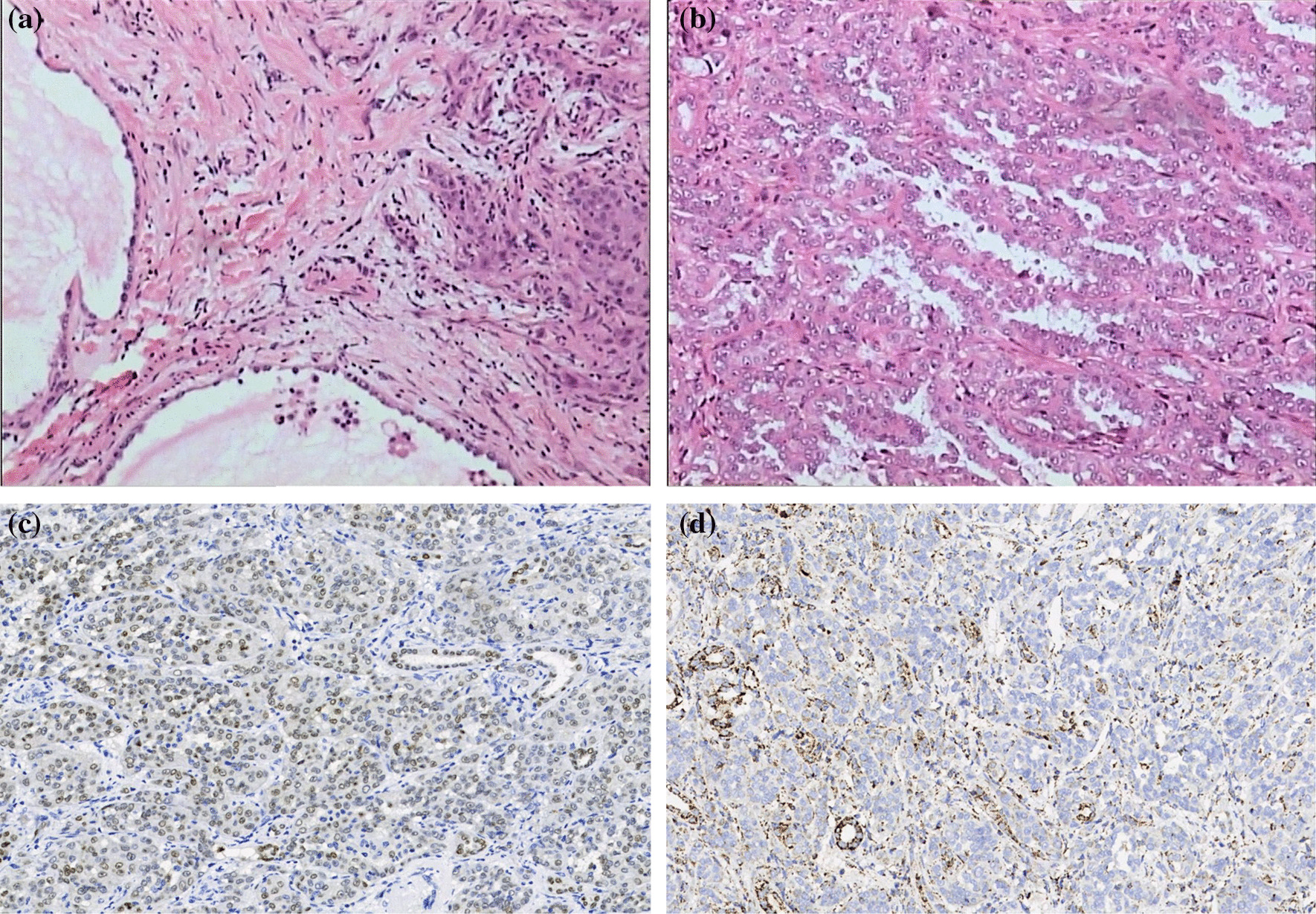
Hematoxylin/eosin staining (**a**) showing that the cyst wall is lined by a monolayer of cuboidal tumor cells. × 100 magnification. Hematoxylin/eosin staining (**b**) showing tumor cells in the solid areas arranged into a papillae pattern, as well as an enlarged nucleus with prominent eosinophilic nucleoli and a peripheral halo × 200 magnification. Immunohistochemistry (**c**) showing positive nuclear expression of PAX8 protein in the tumor cells × 200 magnification. Immunohistochemistry (**d**) showing the absence of Fumarate hydratase protein in the tumor cells; additionally, the nonneoplastic renal cells that served as the internal control showed granular positive expression in the cytoplasm. × 200 magnification

A 60-year-old Asian male who complained of hematuria for 20 days was admitted to the hospital. Ultrasonography showed an extremely hypoechoic mass at the lower pole of the left kidney, enlargement of the right kidney, and multiple hypoechoic areas of the right kidney (cysts and space-occupying lesions were considered), and further examinations were recommended. Subsequent contrast enhanced Computer Tomography (CT) showed that the volume of the right kidney was increased, with a patchy low-density shadow with infiltrative growth inside that had a significantly lower signal intensity than the renal cortex; the possibility of collecting duct carcinoma or lymphoma was considered. Enlarged perirenal and retroperitoneal lymph nodes were also seen (Fig. 1), along with bilateral renal cysts, calcification of the left renal cyst wall, and bilateral kidney stones. Eight years prior, ultrasonography showed a complex renal cyst in the right kidney, and no treatment was administered at that time. The patient had a history of hypertension for 10 years, and his blood pressure was well controlled by oral enalapril 10 mg daily. He had a healthy son and denied a family history of genetic mutations. The other laboratory tests showed no other abnormal findings. Laparoscopic radical nephrectomy of the right kidney was performed at this time, and the postoperative specimens were submitted for pathological examination. Grossly, a mass in the middle and upper poles of the right kidney was seen, with a size of 65 × 35 × 20 mm. The mass broke through the renal capsule and invaded the perirenal adipose tissue. The mass contained cystic and solid regions and was grayish red. The focal area was yellowish, and the texture was moderately hard. Microscopic examination showed that the cystic areas of the tumor were lined by a monolayer of tumor cells (Fig. 2a). The tumor cells in the solid areas were arranged into papillary shapes, glandular tubes, nests and sheets. The tumor cells were enlarged in size and irregular in shape. The cell boundaries were unclear, and the cytoplasm was eosinophilic or slightly transparent. The nucleus was enlarged and atypical, with a significant eosinophilic nucleolus and peripheral halo (Fig. 2b). Abnormal mitoses were clearly seen. A small amount of lymphocyte infiltration could be seen in the stroma. There was no definite bleeding or necrosis. Cancer metastasis was observed in 2 renal hilar lymph nodes. Immunohistochemistry (IHC) showed CK, EMA, PAX8 (+) (Fig. 2c), CD10 focal (+), CK7, Vim, FH (−) (Fig. 2d), and Ki-67 80% (+). Due to the negative expression of fumarate hydratase (FH) protein in a renal tumor that was morphologically similar to fumarate hydratase-deficient renal cell carcinoma (FH-deficient RCC), corresponding molecular pathological tests were performed, and the results showed an FHp.R233H (arginine > histidine) germline mutation (inactivation mutation). The postoperative pathological diagnosis was fumarate hydratase-deficient renal cell carcinoma of the right kidney, T3aN1M0.

## Discussion

FH-deficient RCC is mainly caused by inactivating mutations in the FH gene located in 1q42.3-q43. The inactivation mutation of the FH gene is caused by the germline mutation of one allele and somatic mutation of another allele. The most common type of mutation is missense mutation. Frameshift mutations, nonsense mutations, insertion/deletion mutations and splice site mutations have also been reported [4]. FH catalyzes the conversion of fumaric acid to malic acid in the tricarboxylic acid cycle. The functional defect caused by the inactivation mutation of the FH gene leads to the accumulation of FH and a decrease in iron levels in cells, which inhibits hypoxia inducible factor (HIF) prolyl hydroxylase and increases the level of intracellular HIF. Thus, the mutation affects the transcription of downstream products, angiogenesis, cell proliferation and tumorigenesis [5].

FH-deficient RCC is highly malignant and can metastasize when the tumor volume is very small. The exact incidence of the disease is unknown, and it is mainly seen in Europe and the United States; conversely, this disease is rare in Asian populations, especially in the Chinese population.

The age of onset of the disease is 10–90 years old, and the average age of onset in Europe and the United States is 20–25 years earlier than that of sporadic RCC in the general population [6]. The main manifestations of such patients were hematuria, osphyalgia and other nonspecific symptoms. The main symptom of this patient was hematuria for 20 days without osphyalgia, abdominal pain or symptoms of urinary tract irritation.

The pathological features of the tumor are usually invasive growth and invasion into the surrounding tissue. Microscopically, the tumor cells are arranged into papillary, tubular, solid or cystic patterns. The histological structure is diverse, similar to that of type II papillary renal cell carcinoma, collecting duct carcinoma, sarcomatoid carcinoma and so on [7]. The tumor cells had a significant eosinophilic nucleolus and peripheral halo. The 2 immunohistochemical biomarkers that show a high correlation with the diagnosis of FH-deficient RCC are FH and S-(2-succino)-cysteine (2SC). Although the 2SC protein was not detected in this study because there is no such antibody in our laboratory, the possibility of FH-deficient RCC was considered due to the loss of FH protein expression in a renal tumor that was morphologically similar to FH-deficient RCC. Then, corresponding gene mutation detection was performed for the patient in this article.

FH-deficient RCC is a renal tumor newly defined by the WHO (5^th^ edition) in 2022 [1] and was named hereditary leiomyomatosis renal cell carcinoma (HLRCC)-associated RCC by the WHO (4th edition) in 2016 [8]. The definitive criterion for the diagnosis of HLRCC is the detection of germline mutations in the FH gene. Hansen *et al.* [9] also summarized some major and minor criteria for diagnosis. The former includes the following: (1) at least one lesion histologically confirmed as multiple cutaneous leiomyoma (CLM); and (2) a family history of HLRCC and at least one minor criterion. The minor criteria are as follows: 1. single solitary histologically confirmed CLM; (2) multiple uterine leiomyomas with severe symptom onset in a patient < 40 years old; and (3) Onset of type II papillary renal cell carcinoma in a patient < 40 years old. Meeting the major criteria is highly suggestive of HLRCC. Meeting the minor criteria should raise a suspicion for HLRCC. If the patient has CLM, we should confirm whether the patient has HLRCC through further examinations to avoid a missed diagnosis. Although the first finding in most patients is CLM, the first finding of this patient was RCC, and no CLM was found in this patient or his children.

In terms of differential diagnosis, although FH-deficient RCC has a variety of histological structures, it can be distinguished from other types of RCC based on a combination of molecular pathology characteristics and immunohistochemical findings. For example, type II papillary renal cell carcinoma is characterized by the amplification of chromosome 7/17. MiTF/TFE family translocation RCC shows rearrangement of TFE3 and TFEB. Germline mutation of the SDH gene and loss of SDH protein expression have been found in SDH-deficient RCC. Clear cell renal cell carcinoma shows the loss of 3P and strong positive expression of CAIX protein on the tumor cell membrane. Collecting duct carcinoma is a high-grade RCC that invades the renal medulla, with histological findings of an obvious fibrogenic reaction of the tumor stroma and no characteristic molecular pathological changes. FH-deficient RCC shows a pathogenic germline FH mutation, with negative FH and/or positive 2SC protein expression, but other subtypes of renal cell carcinoma show positive expression of the FH protein, with no inactivated mutation of the FH gene.

In terms of treatment, FH-deficient RCC is highly invasive, and metastasis can occur when the tumor is less than 3 cm [10]. After diagnosis, radical nephrectomy plus lymph node dissection is preferred. Targeted drug therapy or immunotherapy may also bring new breakthroughs in the treatment of these patients. Laparoscopic radical nephrectomy of the right kidney was chosen in combination with postoperative targeted drug therapy and immunotherapy for this patient.

In terms of follow-up monitoring, we recommend that family members at risk of the disease undergo the following assessments:

1. A thorough dermatological examination every other year starting at 10 years of age to assess the existence and development of CLM.

2. Annual gynecological examinations for women starting at 20 years of age to assess the growth of uterine leiomyomas.

3. Renal monitoring for patients with FH gene mutations according to the following recommendations:

- 3.1 Yearly MRI scans starting at the age of 8–10 years.
- 3.2 If renal cysts are detected, closer monitoring is indicated, as follows:
- 3.2.1 during the 1st year: at 3, 6, and 12 months after the detection of the cysts, if no solid nodules appear.
- 3.2.2 during the 2nd–4th years: every 6 months, if no solid nodules appear.
- 3.2.3 from the 5th year and onward: yearly MRI scans.
- 3.3 If solid nodules are detected, brain MRI and whole-body fluorodeoxyglucose-positron emission tomography (PDG-PET) should be for staging (repeat 1 × after 3 months) [11, 12].

After the patient in this case was diagnosed, his son and grandson were tested for the FH gene, and no mutations were found. The patient was treated with targeted drugs: oral administration of sunitinib 50 mg once a day. The patient took the medicine for 4 weeks and then stopped for 2 weeks. The tumor progressed, and bone and liver metastasis occurred half a year later. The treatment was then changed to oral axitinib 5 mg twice a day and toripalimab 240 mg every 21 days via an intravenous drip. At present, the patient is in stable condition, and there has been no progression of the metastases.

## Conclusion

FH-deficient RCC is a very rare renal tumor, and the definitive criterion for diagnosis is the detection of germline mutations in the FH gene. Early genetic testing and renal surveillance are of significant importance in high-risk family members. Moreover, FH-deficient RCC is highly malignant and metastasizes early. Therefore, fully understanding the disease, enabling detection and diagnosis and providing treatment are particularly important.

## Data Availability

PubMed was used as a source of information, using the search terms fumarate hydratase AND deficient AND renal cell carcinoma.

## Abbreviations

FH: Fumarate hydratase
RCC: Renal cell carcinoma
HLRCC: Hereditary leiomyomatosis renal cell carcinoma
HIF: Hypoxia inducible factor
CLM: Cutaneous leiomyoma
CT: Computed tomography
FDG: Fluorodeoxyglucose
PET: Positron emission tomography
HE: Hematoxylin/eosin
IHC: Immunohistochemistry
